# *LTRsift*: a graphical user interface for semi-automatic classification and postprocessing of *de novo* detected LTR retrotransposons

**DOI:** 10.1186/1759-8753-3-18

**Published:** 2012-11-07

**Authors:** Sascha Steinbiss, Sascha Kastens, Stefan Kurtz

**Affiliations:** 1 Center for Bioinformatics, University of Hamburg, 20146 Hamburg, Bundesstrasse 43, Germany

**Keywords:** Software, ERV, LTR retrotransposons, Classification, Postprocessing, Prediction, Annotation, User interface

## Abstract

**Background:**

Long terminal repeat (LTR) retrotransposons are a class of eukaryotic mobile elements characterized by a distinctive sequence similarity-based structure. Hence they are well suited for computational identification. Current software allows for a comprehensive genome-wide *de novo* detection of such elements. The obvious next step is the classification of newly detected candidates resulting in (super-)families. Such a *de novo* classification approach based on sequence-based clustering of transposon features has been proposed before, resulting in a preliminary assignment of candidates to families as a basis for subsequent manual refinement. However, such a classification workflow is typically split across a heterogeneous set of glue scripts and generic software (for example, spreadsheets), making it tedious for a human expert to inspect, curate and export the putative families produced by the workflow.

**Results:**

We have developed *LTRsift*, an interactive graphical software tool for semi-automatic postprocessing of *de novo* predicted LTR retrotransposon annotations. Its user-friendly interface offers customizable filtering and classification functionality, displaying the putative candidate groups, their members and their internal structure in a hierarchical fashion. To ease manual work, it also supports graphical user interface-driven reassignment, splitting and further annotation of candidates. Export of grouped candidate sets in standard formats is possible. In two case studies, we demonstrate how *LTRsift* can be employed in the context of a genome-wide LTR retrotransposon survey effort.

**Conclusions:**

*LTRsift* is a useful and convenient tool for semi-automated classification of newly detected LTR retrotransposons based on their internal features. Its efficient implementation allows for convenient and seamless filtering and classification in an integrated environment. Developed for life scientists, it is helpful in postprocessing and refining the output of software for predicting LTR retrotransposons up to the stage of preparing full-length reference sequence libraries. The *LTRsift* software is freely available at http://www.zbh.uni-hamburg.de/LTRsift under an open-source license.

## Background

Large portions of eukaryotic genomes are repetitive, that is, the sequences in question appear in more than one genomic location. Repetitive DNA (in the scope of this paper also referred to as repeats) can be further subdivided into a hierarchy of categories, the most general of which are simple repeats – for example, satellite DNA or telomeres – and interspersed repeats, for example, transposable elements (TE). Interspersed repeats are abundant in the genomes of many higher organisms. For example, about 46% of the human genome [1] and 38% of the mouse genome [2] consists of interspersed repeats, most of which are transposons. These, in turn, are further characterized by their transposition mechanism: class I transposons or retrotransposons replicate via an RNA intermediate, while class II transposons or DNA transposons replicate via a DNA intermediate [3,4].

**Figure 1:**
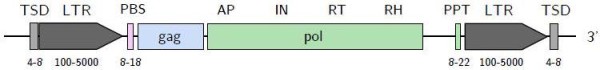
**Structure of a typical long terminal repeat retrotransposon.** Adapted from [5]. AP:Aspartic protease; IN: integrase; LTR: long terminal repeat; PPT: polypurine tract; RH: RNase H; RT: reverse transcriptase; TSD: target site duplication. The numbers below the illustration denote typical lengths of the respective component. This example is of the *copia*-like superfamily, as it shows the IN-RT-RH domain order.

An important order of retrotransposons are LTR retrotransposons, which are less common in animals, but the predominant order in plants. Similar in structure to retroviruses (Figure 1), they show long terminal repeat (LTR) sequences at their flanks, with adjacent small direct repeats resulting from target site integration (target site duplications or TSD). The sequence between the LTRs typically encodes polyprotein genes, for example, *gag* and *pol*, containing structural proteins for an intracellular replication compartment as well as enzymatic functions required to perform the reverse transcription and integration process. This includes an aspartic protease (AP), reverse transcriptase (RT), RNase H (RH), and integrase (IN) function.

Additionally, several other structural elements are required for successful transposition. These include the primer binding site (PBS), which is essential as the start point for reverse transcription [6-8]. Typically the PBS is 8 to 18 base pairs long and expected to be found directly downstream of the inner 5’ LTR boundary. In this region of 8 to 18 nucleotides, it is also highly complementary to the 3’ region of a transfer RNA of the host organism. Another important feature is the polypurine tract (PPT), needed as a primer for plus-strand DNA synthesis. The A/G-rich polypurine tracts vary in length and are usually in the range of 8 to 22 bases [8]. Often a U-rich section (the so-called U-box) can be found just upstream of the PPT [9].

The presence of these distinctive structural features have led to the development of various software tools using these features as markers to identify potential LTR retrotransposon insertions (called candidates in the scope of this paper) [10-14]. These tools do not use any external reference sequence, an approach called de novo identification. The rationale behind this approach is that transposon sequences are typically species-specific and a purely homology-based identification approach is not guaranteed to be successful. Instead, for transposon annotation in newly sequenced genomes with no or only few related and annotated genomes, a *de novo* approach is required. Some tools also detect internal features of the candidates and exploit their occurrence to improve the candidate detection results [13] or output the feature annotations for further analysis [5].

For about a decade, *de novo* software tools have been in use, delivering useful results in a variety of detailed studies covering LTR retrotransposons in insect [5,15,16], crustacean [17], mammalian [18,19], avian [20], metazoan [21] and plant [22,23] genomes. In these studies, the prediction is followed by several postprocessing steps to clean up the result set and to infer additional information from the predicted candidate sequences and features. For example, it is reasonable to separate all candidates from the analysis set which do not satisfy a given set of rules (for example, presence of significant open reading frames or profile hidden Markov models (pHMM) domain hits) to identify and discard potential false positives [5,12,21].

Furthermore, it is desirable to classify each candidate according to a hierarchical schema consisting of classes, subclasses, orders, superfamilies and – on the lowest level – individual families. We will use these terms as defined in the classification scheme proposed by Wicker and colleagues [4]. This schema has been widely accepted despite some initial discussions regarding its consistency [24] and originality [25].

It has to be noted that in this paper we will focus on the order of LTR retrotransposons from the retrotransposon class (class I), as such elements are the result of the *de novo* identification tools. However, if more general repeat detection approaches are used, a preclassification on a higher level is possible using existing software, for example, TEclass [26] on the class level, or REPCLASS [27] up to the superfamily level.

In the LTR retrotransposon order, several superfamilies have been established, for most of which membership can be determined by the order of protein domains in the coding region. For example, *copia*-like elements (IN-RT-RH configuration) are distinct from *gypsy*-like elements (RT-RH-IN configuration). Thus if protein domain locations are known, subfamily assignment is straightforward. If protein domain locations are not known, pHMM-based approaches using superfamily-specific reference sequences have proven to be successful [21].

A more fine-grained classification groups the candidates into putative families. Most studies perform the family classification using either sequence-based clustering of the inter-LTR region of the predicted candidates [15-17] according to fixed similarity thresholds (for example, the 80-80-80 rule [4]), by inferring family membership from phylogenies [19,20,28] or by using graph clustering algorithms based on stochastic flow [21], a method that does not require any arbitrary similarity threshold. Other methods – though not used in an LTR retrotransposon context - follow an aggregation procedure based on pairwise distances [29]. Another approach is to obtain sets of similarity-based clusters per feature (LTR, PBS, PPT, protein domains and so on), then to combine them adhering to the principle of cluster compatibility [5], arriving at final family assignments. This approach makes use of biologically relevant sequence information only and is expected to work for families containing candidates with partially deleted or mutated internal regions. A case study of this method on *Drosophila melanogaster* candidates predicted by *LTRharvest*[14] and annotated using *LTRdigest*[5] showed that this approach recovers the majority of the known families in this species, with well-reproduced reference sequences [5].

However, putting this family classification scheme into practice is rather complex and time-consuming. A possible approach would be to augment the tabular file output of *LTRdigest* with cluster number data using third-party matching and/or clustering tools (for example, Vmatch [30]), then open the augmented table in a spreadsheet software (for example, Microsoft Excel or OpenOffice Calc) and to iteratively sort the candidates by cluster number, inspecting candidates with identical cluster number sets and marking compatible groups as putative families in the process. Removal of uninteresting candidates or false positives is in this case only a matter of deleting the corresponding rows in the spreadsheet. While this manual approach is feasible (but tedious) for smaller non-mammalian genomes (713 candidates and 25 features in the *D. melanogaster* example from [5]), it does not scale too well with growing candidate sets. This is due to both the long run times of sorting rows in such software and the presentation and/or visualization of results getting more and more difficult to follow with increasing candidate and family numbers. Another important issue is the reproducibility of such a manual approach when prediction runs are repeated, for example with modified parameters.

Since the classification as proposed is an algorithmically well-defined problem, an alternative would be a purely automatic software implementation of the approach, for instance using a scripting language. However, a human expert often would like to inspect and improve the resulting family assignments, not only to get an impression of how the individual families look, but also to verify that no two families were inadvertently joined, or that one family could be split up into two at a second glance. Later steps then would include matching the sequence of a family representative to a set of reference sequences to identify relatives to known families, or to create multiple sequence alignments (MSA) of family members determining variations across family members. As an alternative to MSAs, the identification and analysis of units called modules has been proposed [31].

The final step, after filtering and classification, is the preparation of a species-specific reference sequence library of full-length LTR retrotransposons. Consisting of one representative sequence per putative family, this library can then be used as a basis for homology-based identification and classification of incomplete, non-autonomous insertions or solo LTRs in the whole genome sequence. This can be done using well-established software, for example, based on *RepeatMasker*[32].

In this manuscript we propose a *de novo* analysis approach of LTR retrotransposons relying on a combination of automatic and manually guided interactive processing of a given dataset. For this approach a comprehensive workbench for LTR retrotransposon candidate postprocessing with the following features is needed: 

• intuitive display of a candidate set (for example, unclassified candidates or putative families) and its properties in a flexible, concise table representation

• hierarchical display of detected features for each candidate, including a linear diagram illustrating the feature locations and orientations

• convenient maintenance of putative families and their members via a drag-and-drop interface

• flexible, extensible filtering and reassignment of candidates based on simple, annotation-based rules

• assisted assignment of candidates to putative families, based on feature sequence matches, single-linkage clustering and joining of compatible candidates

• automatic selection of candidates suitable for a reference library

• automatic annotation of candidates with matches to a reference library

• input and output of library sequences and annotations in standard output formats (GFF3 [33] and FASTA) to ensure interoperability with external preprocessing and/or postprocessing software.

To address this need we have developed *LTRsift*, an open source graphical software tool implementing these features. As *LTRsift* is based on a larger software suite that also includes *LTRharvest*[14] and *LTRdigest*[5], a typical and complete use case will likely include all three tools.

This paper is structured as follows: after familiarizing the reader with the interface and usage of *LTRsift*, we present two case studies showing how the software can assist a researcher in *de novo* analyses of complete genomes. First, we perform the *Drosophila melanogaster* analysis from the *LTRdigest* paper [5], exemplifying the use of the software. In a second use case, we briefly describe an analysis from scratch using *LTRsift* on a mammalian genome, specifically the gray short-tailed opossum (*Monodelphis domestica*) genome. We show that *LTRsift* scales for such larger data sets yielding some interesting results. We give a comprehensive discussion of the result and finally conclude with some remarks on the usability of the software.

## Implementation

This section describes the use of the *LTRsift* software and the design of its user interface. We explain the import of input data into the software and the initial preprocessing. Then we describe the components of the *LTRsift* window and show what information they depict and how they allow curation of the candidate sets. We show how to define filtering rules, possibly incorporating all the information given in the annotation file. Finally, we give some examples for such rules.

### Working with data

All data handled by *LTRsift* are organized as projects. A project is a collection of data, consisting of the candidate annotation, the sequence set the annotations are based upon (given as an external encoded sequence file in the *GtEncseq* format [34] that is produced by software coming with *LTRsift*), and various metadata (such as the currently open tabs, parameter sets for classification/matching, and so on). A user can create a new project by selecting the ‘New’ entry from the ‘File’ menu. A series of dialog windows then guides the user through the steps of specifying all components of the project. The first step is to specify a project (file) name as well as the initial annotation and sequence inputs. This is done by choosing the corresponding GFF3 and *GtEncseq* files from disk. The GFF3 annotations have to satisfy the following basic requirements: 

• candidates must be of the Sequence Ontology [35] type ‘LTR_retrotransposon’,

• additional features (for example, ‘protein_match’, ‘primer_binding_site’, ‘RR_tract’) must be children of this root type, and

• the GFF3 sequence identifier for all features must start with ‘seq*X*’, where *X* is the sequence number (0-based) of the corresponding sequence in the associated genome, for example, ‘seq3’ references the fourth sequence in the encoded input index.

The GFF3 output produced by *LTRharvest* and *LTRdigest* satisfies these requirements. More information about the input data formats can be found in Additional file 1, Section 1.2.

If the user chooses to compute the matches required for the clustering process, then the second step is parameterization of the matching and clustering parameters. *LTRsift* utilizes the sensitive and efficient sequence comparison software LAST [36] to calculate matches if feature sequences are longer than 80 nucleotides on average.

If the user has chosen to perform the automatic classification at this point, the next step is to specify which features should be used as the basis of classification, that is, for which features in the annotation cluster numbers should be compared. In addition, a prefix for automatically assigned family names can be given. Since the notion of most complete candidates depends on the deviation of candidates from the group median in terms of LTR and whole-candidate length [5], deviation thresholds can be stated as well. All settings can be reviewed and corrected if necessary before starting the actual import and preprocessing run. This process finally delivers a new project file, which can later be opened at any time using the ‘Open’ entry from the ‘File’ menu. This allows the user to continue a previously interrupted session.

### User interface

**Figure 2:**
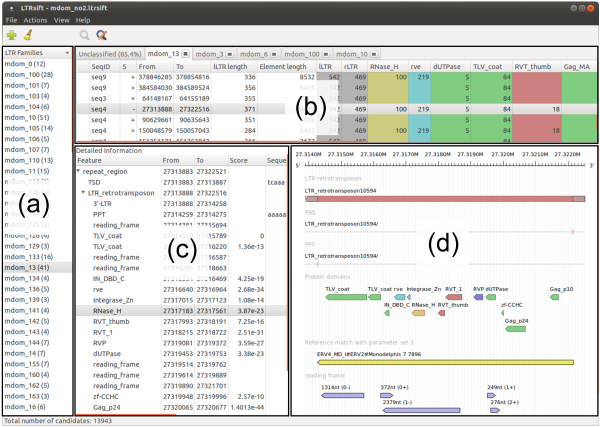
**Screenshot of the *LTRsift* main window.** (**a**) Putative family list, (**b**) candidate list, (**c**) candidate details, (**d**) candidate visualization. The currently loaded project contains 13,943 candidates from the *Monodelphis domestica* genome, with the currently selected candidate showing a full set of detected features (PBS, PPT, protein domains). ORF detection and reference matching have been performed. Additional details, such as PPT and PBS sequences, Pfam IDs and so on, are available by scrolling to the right in (**c**). The graphical representation in (**d**) depicts the retrotransposon (red) with PPT and PBS as small lines in the two tracks below. The next track shows protein domain matches, coded in different colors. Here integrase domains are depicted in blue, reverse transcriptase domains in red, protease domains in purple, RNase H domains in gold, and any other domains in green. The RNase H domain is marked in red because it has been selected in the candidate detail list. The reference match in the track below (shown in yellow) spans the interior region of the candidate completely, suggesting that it likely is a full-length element. The bottom track shows open reading frames in blue color. LTR: long terminal repeat; ORF: open reading frame; PBS: primer binding site; PPT: polypurine tract; TSD: target site duplication.

After opening a project file, its content appears in the main window of *LTRsift*. The main window is subdivided into four main components (Figure 2). The family list (Figure 2a) shows the current list of putative families, identified by their names. Names are automatically assigned whenever a new family is created by the classification algorithm. However, a family name can be edited at any time.

A double click on a family opens a new tab holding the candidate list comprising the family (Figure 2b). Each tab, labeled with the family name, shows all member candidates of a given family, with columns specifying sequence, strand, location, LTR and element lengths, and cluster numbers for all detected features according to a color code. Colors are user-configurable via a style definition file. By default LTR, *gag*-associated domains, AP domains, RT domains, RH domains and IN domains, as well as PPT and PBS are displayed in different colors. Candidates which could not be placed unambiguously in one of the families remain in an ‘unclassified’ tab, as do candidates with no cluster numbers (singletons). The rows making up the candidate list can be sorted according to the values in any column. Moreover, individual columns can be hidden to improve legibility on screens with low horizontal resolution. Candidates can be moved from one family to another by dragging and dropping the respective row in the candidate list. Whenever candidates are deleted from families, they are added to a project-wide list of unclassified candidates. Candidates deleted from the list of unclassified candidates will be removed from the project entirely.

Clicking on an entry in the candidate list displays additional detailed information. In particular, a hierarchical tree representation of the candidate features (Figure 2c) and a linear visualization of the candidate and its components (Figure 2d) are displayed. The latter depicts the candidate together with its genomic location, most likely reading direction and internal features, intuitively spread out into separate tracks reminiscent of the representation used in a genome browser such as Ensembl [37].

### Augmenting annotations with additional data

*LTRsift* does not only allow displaying results of the automatic classification, but can also perform additional operations which add extra information to the annotations stored in the project. One possible augmentation consists of detecting the longest open reading frames (ORFs) inside candidates. The ORFs are added to the candidate annotations as ‘reading_frame’ features. For cross-referencing the *de novo* results with custom sequence data, *LTRsift* also allows users to match candidates against external reference sequences by calling BLAST [38] as an external matching tool. The parameter sets used for all matching runs are numbered and stored in the project both for documentation purposes and for simplifying multiple runs with varying but similar parameters. *LTRsift* allows flexible filtering of candidate sets, based on filtering rules defined in a simple programming language (see next section for more details). Candidates which do or do not satisfy the filtering conditions (or boolean combination thereof) can be either unclassified or moved to a new family entry created on the fly. These postprocessing steps can either be performed on all candidates, on all candidates in a family, or on an arbitrary selection of candidates.

### Filtering rule definitions

Besides the described presentation of candidate sets, *LTRsift* allows for flexible filtering of candidates based on their internal structure or external data. This is done by evaluating each candidate with regard to a given chain of annotation-based rules. Each rule specifies one aspect of the candidate which is satisfied or not, expressed via a boolean value (*true* or *false*). We call these rules predicates. For example, the presence of detected protein domains is a predicate, as is the property of being *Copia*-like (or *Gypsy*-like, for that matter). The predicate is captured in a rule returning the appropriate boolean value.

**Figure 3:**
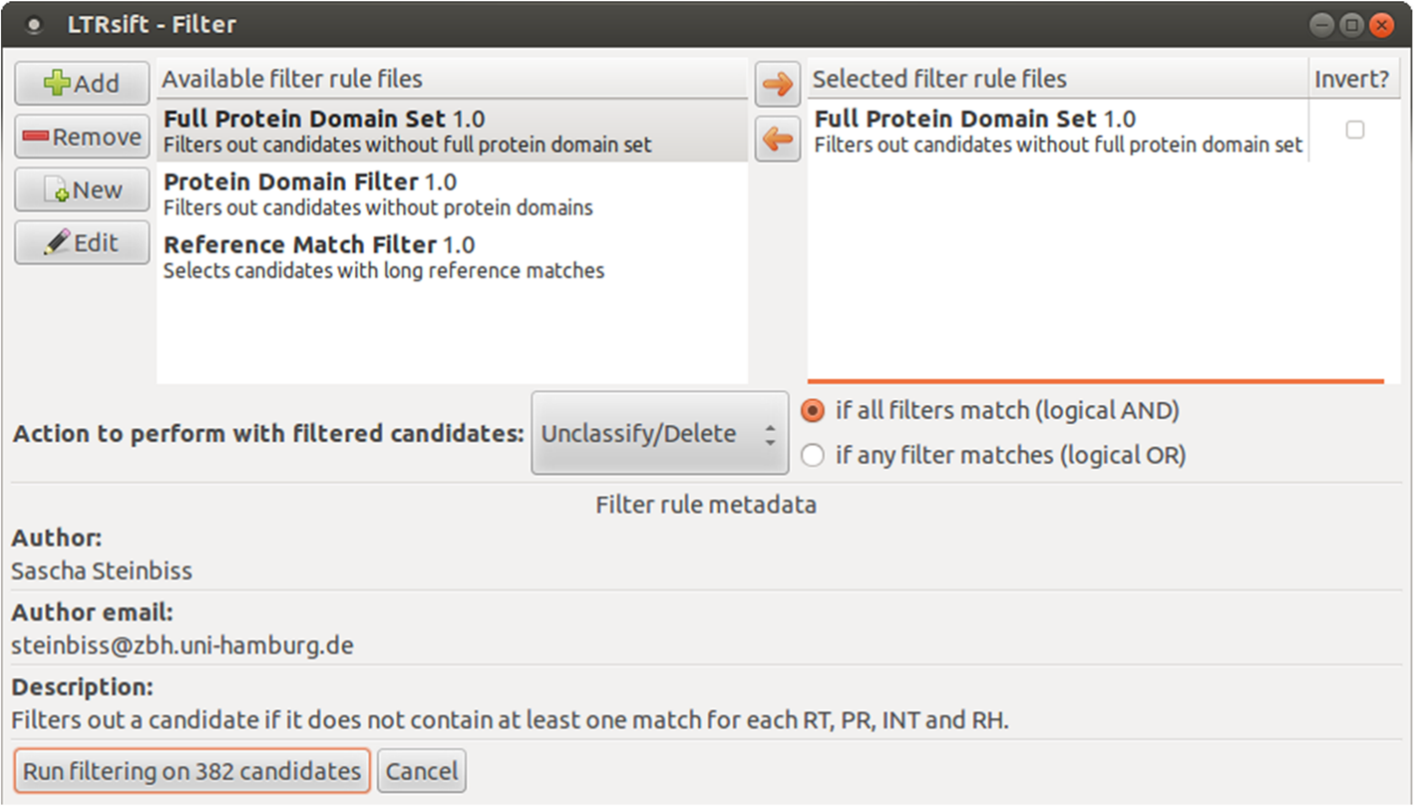
**Screenshot of the *LTRsift* filter selection dialog.** The left side of the dialog shows the filtering rules added to the project and available to be used. The right side shows the filtering rules to be applied in the current filtering run. The checkbox next to each rule allows the user to negate it. This dialog is set to unclassify or delete all candidates not passing the filtering step - in this case, this means all candidates that do not contain protein domains and no long reference matches. The buttons on the left allow adding rules to the project and removing them again. Moreover rules can be edited directly from within *LTRsift* in a simple built-in text editor, avoiding the need to locate and open them in a separate text editor. Clicking the button on the lower left starts the filtering process.

When a filtering run is started, candidates are selected according to rules, keeping a candidate in its original family if the evaluation returns the value *false*. If it returns the value *true*, the desired behavior can be selected by the user. Either the candidate in question is taken out of a putative family and put back into the unclassified set. This means that filtered candidates already in the unclassified set are deleted from the project altogether. Alternatively the candidate in question can be assigned to a separate family, newly created for candidates which were filtered out in this run. Which one of these options is used can be selected in the filtering dialog (Figure 3). Rules are chainable: if more than one rule is given, *LTRsift* allows their combination by requiring that all of them must be *true* to filter out a candidate (boolean AND), or that it suffices to have one of them evaluate to *true* (boolean OR).

The rule is stored in a text file which is interpreted by the software when added to the project. Each filtering rule contains a set of metadata, such as its author contact, description, and version to ease distributability and reproducability of results (see Additional file 1, Section 1 for more details).

As the filtering rules are not built into *LTRsift* itself, but rather described in Lua, a powerful but simple programming language [39], the filtering functionality is extensible which gives a user a maximum amount of flexibility. We will now show example filtering rules to illustrate how annotation data are accessed.

#### Filtering by protein domain presence

As the protein domain coding sequences are the main basis for the matching and clustering steps leading to family assignment, a common task is to remove all candidates which do not contain at least one domain hit.

This task can be solved by a rule implemented in a function named filter. This function has access to a representation of each candidate in the form of a directed acyclic graph in which nodes represent individual features (for example, LTR, TSD, PBS, …) and edges represent ’part of’ relationships between such features. The latter indicate, for instance, that an LTR is part of an LTR retrotransposon, which in turn is part of a repeated region.

The actual rule defined in the programming language Lua looks as follows:

function filter(candidate_node)

gfi = gt.feature_node_iterator_new (candidate_node)

node = gfi:next()

while not (node == nil) do

if (node:get_type() == "protein_match") then

return false

end

node = gfi:next()

end

return true

end

The rule evaluates whether the candidate contains a ‘protein_match’ feature. The traversal stops and returns *false* once a node with the type ‘protein_match’ is found. If no such node is found, *true* is returned. This is the case when node becomes nil, indicating that all children have been examined without breaking the loop.

Besides the type, the following additional data are stored in each node and can conveniently be queried from a filtering rule: 

• the sequence region (for example, chromosome, contig, and so on) the feature is located on,

• location of the feature on that region in terms of start and end position (1-based),

• strand (forward/reverse),

• a numeric score value (the meaning of which depends on the tool which produced this value, for example, an E-value),

• a set of key-value pairs containing arbitrary named attributes (for example, feature name/ID, anticodon for PBS-binding tRNAs, Pfam ID for matching pHMMs, and so on).

#### Filtering by match coverage

**Figure 4:**
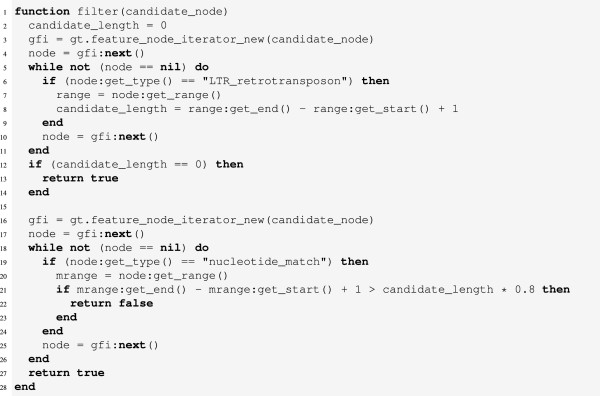
**Source code (in the programming language Lua) of the filtering rule for selecting/filtering candidates according to reference match coverage.** The function computes the lengths of the candidate and reference matches contained in the candidate. If the length of at least one reference match exceeds 80% of the candidate length, the function returns *false*, otherwise *true*.

Another use case for the filtering component is to separate candidates with high local sequence similarity to a reference sequence set from those that do not contain such similarities. To give an example, a filtering rule which filters out all candidates not matching a reference sequence over at least 80% of their length is easily implemented (Figure 4).

In the first step (lines 2 to 11), the length of the candidate is calculated from the start and end positions of the node in this connected component with the type ‘LTR_retrotransposon’. We only store the length of the last occurrence of such a node because in a valid annotation there is only one such node per connected component. The length is stored in the variable candidate_length. If no “LTR_retrotransposon” node was found, we are not looking at an LTR retrotransposon element (lines 12 to 14). This case can occur when filtering annotation files in which LTR retrotransposon annotations occur besides other annotations, e.g. gene annotations, which this rule is designed to ignore. The second step then iterates over all features again, comparing the lengths of the matches with 80% of the candidate length (line 21) and returns the appropriate value in the process (lines 16 to 27).

### Additional command-line tools

For very large data sets or for scripted processing (for example, in an automated pipeline), the filtering and clustering functionality is also accessible using command-line tools, which are part of the *GenomeTools* package.

### Data export

Candidate sequences (in FASTA format) and candidate annotations (in GFF3 format) can be exported to files. *LTRsift* supports exporting all candidates in the whole project into a single file as well as exporting the members of one or more specific families into one or multiple separate files. This makes it easy to prepare sequence sets for subsequent external analysis, for example, multiple sequence alignment.

### Software requirements

*LTRsift* is intended for use on UNIX-like operating systems, like Linux, Mac OS X or the BSD family of operating systems. The GUI is built upon the GTK+ version 2 toolkit for creating graphical user interfaces [40,41], a wide-spread library component which is already being used as the basis of many popular free software packages like the GIMP image editor [42] or the GNOME desktop environment [43]. GTK+ is included in the majority of currently available Linux distributions.

The necessary components for parsing and handling both sequence and annotation data are provided by the *GenomeTools* genome analysis library [44], freely available from http://genometools.org. For drawing the schematic illustration of each candidate, the *AnnotationSketch* engine [45] is used, which is based on the Cairo 2D vector graphics engine [46] for rendering. Again, Cairo is a standard library very likely to be present on a recent graphical UNIX or Linux machine. It can be installed on Mac OS X using one of several package managers, for example, Fink [47].

As an external component to compute initial matches for clustering, the LAST software [36] is used and must be installed on the system. Likewise, for reference matching the BLAST software (version 2.2 and higher) must be present in order to use this *LTRsift* feature.

Also, we provide a statically linked version of *LTRsift* for the Linux platform which does not require any pre-installed version of the *GenomeTools* shared library.

### Results

To illustrate the use of *LTRsift* in *de novo* LTR retrotransposon analysis efforts, we performed example analyses of the *Drosophila melanogaster* (fruit fly) and *Monodelphis domestica* (gray short-tailed opossum) genomes.

### Results of the *Drosophila melanogaster* analysis

We utilized *LTRsift* to semi-manually curate and postprocess putative LTR retrotransposon families and potential full-length members in the *Drosophila melanogaster* release 5.8 genome [GenBank:AE014134, GenBank:CM000456, GenBank:AE013599, GenBank:CM000457, GenBank:AE014296, GenBank:CM000458, GenBank:AE014297, GenBank:CM000459, GenBank:AE014135, GenBank:AE014298, GenBank:CM000460] from scratch, detected using *LTRharvest* and *LTRdigest*. These were parameterized with range, similarity and offset constraints for the detection of LTR pairs, and with tRNA sequences as well as profile HMMs to annotate the internal region. These parameters were identical to the parameters used in previous publications [5,14]. We refer to Additional file 1 for more details on the parameterization of these two external tools. For both the initial detection and the subsequent *LTRsift* analysis steps described below, an Intel Core 2 Duo system (2.4GHz, 4GB RAM, Ubuntu Linux 12.04) was used.

The encoded genome sequence and the sorted GFF3 file were added to the project using the guided project creation dialog. Then matching and clustering of all feature sequences was performed in a matter of minutes, keeping the default parameters in the appropriate dialogs, with the exception that the query step size (LAST parameter -k) was increased to 10 to speed up the process. We required matches to span 80% of the shorter sequence and 30% of the longer sequence. For the classification step, all detected features were used as evidence in the group joining process by selecting all of them in the classification dialog. As deviation thresholds for putative full-length candidate detection, an LTR length deviation of 50 bp and a candidate length deviation of 200 bp were set. No filtering was done at this time.

After creating the project, the initial classification resulted in 359 candidates being split up into 48 putative families, appearing in the putative family list. The remaining 354 (49.6%) remained unclassified. Afterwards we discarded all families with less than three members by using an automatic *LTRsift* feature which selects such families and offers to delete them. Of the original 48 families, 37 remained. In the next step, each of the putative families was individually opened in a new tab and their members were inspected one after the other by examining the data displayed in the candidate detail list and the linear visualization. Special attention was paid to the candidate length and the length of the LTRs they contain as well as their feature composition. Candidates which obviously lacked features present in the majority of the members of their family were discarded by deleting them in *LTRsift*, placing them back into the group of unclassified candidates. This was particularly the case when a length aberration coincided with the loss of a common protein domain. This situation was regarded as evidence for a deletion inside the candidate in question. After inspecting all groups in this way, we had a closer look at the previously unclassified candidates by opening the respective tab and manually joined another 134 of them into four additional groups, again looking at their details. In addition to cluster compatibility, this joining was based again on element and LTR length as well as specific features such as the presence of ORFs and their lengths. For this manual approach, new empty families were created using the *LTRsift* GUI and the respective member candidates were moved into them using the drag-and-drop functionality.

To evaluate which existing families our *de novo* identified families correspond to, we used the reference sequence matching function in *LTRsift* to compare all candidate sequences with the reference sequence set of LTR retrotransposons used in our previous work [5], containing 61 sequences. After matching the reference sequences to the candidates from within *LTRsift* using BLASTN (E-value threshold: 0.01), the results are displayed as an extra track in the candidate visualization. That is, in the delivered image the matched candidate region is covered by a separate feature, while the label of the feature describes the matched region on the reference. We examined our putative families for consistency according to the match features and assigned names which incorporate the recovered reference family (for example, the group corresponding to the existing *mdg3* family was called ‘dmel_1 (mdg3)’).

With little effort, we were able to recover 28 of the 61 known families as defined by the reference sequence set (Table 1). In one case (the family named *412*), we obtained two automatically derived putative families for one reference family due to the LTR sequences of the candidates ending up in different clusters, breaking their compatibility. The *roo* family proved to be a difficult family to recover due to the presence of only short protein domain matches within their members. However, joining them on the basis of the LTR sequences alone allowed us to obtain a putative family of 94 candidates, though not all of them appeared to be full-length. Another group difficult to separate was a group of elements belonging to the *Stalker*/*Stalker2*/*Stalker3T*/*Stalker4* families. Members of all of these were clustered into one large family, possibly due to the high similarity of their coding sequence.

**Table 1:** **Results for the *Drosophila melanogaster* use case**

**Assigned family**	**Reference family**	**Number of candidates**
dmel_1	mdg3	12
dmel_3	opus	18
dmel_4	copia	24
dmel_5	springer	6
dmel_6	Burdock	16
dmel_7	diver	8
dmel_8	HMS-Beagle	10
dmel_9	Tirant	19
dmel_10	Tabor	4
dmel_11	Quasimodo	14
dmel_12	Transpac	9
dmel_14	flea	16
dmel_17	invader2	8
dmel_17_2	invader3	7
dmel_18	Max-element	4
dmel_22	3S18	6
dmel_24	McClintock	4
*dmel_26*	*Stalker*	*25*
dmel_32	17.6	18
dmel_33	412	17
dmel_34	412	9
dmel_36	Idefix	5
dmel_39	rover	5
dmel_46	micropia	3
newfam_0	blood	25
newfam_29	HMS-Beagle2	4
newfam_40	297	20
manual	gypsy4	7
manual	mdg1	17
manual	roo	94

This table lists the putative families as assigned during our semi-automatic evaluation run on the *Drosophila melanogaster* genome (left column). The center column shows the name of the known family represented by that putative family, obtained from matching of the candidate sequence against a reference sequence set. The rightmost column lists the number of candidates in the respective family. The *dmel_26* group (matched to various *Stalker* sequences) was not counted as recovered due to the multitude of non-unique matches to multiple references. Families with the *newfam* prefix were obtained by re-running the classification algorithm on subsets of the unclassified candidate set. Finally, families marked as *manual* were derived non-automatically.

Seven additional putative families were found which could not be uniquely matched to any reference family. The majority of these consist only of three to five candidates with widely varying element and LTR lengths. These candidates are linked only by one protein domain or LTR sequence match and show short ORFs only. In some of them, spurious short matches to the *Dm88* and *GATE* families were found in non-coding areas of these candidates.

We have included the annotation files generated by *LTRsift* for this analysis as Additional file 2.

### Results of the *M. domestica* analysis

By contrast, the goal of this analysis was to confirm that the *LTRsift* software can handle input data of a scale likely to be produced in *de novo* LTR retrotransposon prediction efforts in large genomes, for example, those of mammals. As an example dataset, we applied *LTRharvest* and *LTRdigest* to the genome of *M. domestica*, the gray short-tailed opossum [48]. It is estimated that 10% of its approximately 3.5 gigabase genome is comprised of endogenous retroviruses (ERV) [49,50]. This has been detected in a homology-based approach searching for protein-coding sequences known from the Repbase database [51], but not incorporating the LTRs as structural features for *de novo* detection.

The *M. domestica* sequence (Broad Institute assembly version MonDom5) [GenBank:CM000368, GenBank:CM000369, GenBank:CM000370, GenBank:CM000371, GenBank:CM000372, GenBank:CM000373, GenBank:CM000374, GenBank:CM000375, GenBank:CM000376] was downloaded from the Ensembl website [52].

The *LTRharvest* and *LTRdigest* runs and the subsequent *LTRsift* analysis were performed on the same Linux standard desktop system equipped with the same hardware as described above. We used a slightly different set of parameters than in the *Drosophila* case because some peculiarities in the fly genome (such as unusual PBS-tRNA binding offsets) are not known for the opossum genome. In addition, we used some extra protein domain pHMMs suitable for mammals (see Additional file 1, Section 3).

The *LTRharvest* prediction with default parameters (see Additional file 1, Section 3) delivered 58,684 candidates. We then utilized the command-line filtering tool to remove all candidates which did not contain any protein domain hits, reducing the number of candidates to 13,944.

This set of candidates was then loaded into an *LTRsift* project and preprocessed. The matches used for clustering were computed with the same settings as in the *Drosophila* case (LAST option -k 10, matches need to span 80% of the shorter sequence and 30% of the longer sequence). Full-length member deviation thresholds were identical as well.

Matching and joining of the 13,944 candidates took about 30 minutes. As a result of the initial classification on the basis of the parameters above, 171 putative families were created containing 2,015 candidates altogether. There were initially 11,929 candidates that were unclassified, and 76 of the 171 initial putative families contained only two members and were again discarded using the respective *LTRsift* functionality. This left 95 remaining families comprising 1,863 candidates.

A look at the 95 remaining putative families reveals that the distribution of candidates across the families is skewed: 937 of the 1,863 candidates are in two putative families, one with 722 members and one with 215 members. The candidates in the smaller one are linked via their LTR and RT clusters only. The linear visualization in *LTRsift* shows that the location of the RT matches widely varies across the family members. Only very short ORFs are present. This may suggest that this family of 215 members is composed of non-autonomous candidates with many mutations. By contrast, the majority of the members of the second, larger putative family of 722 members contain a full set of protein domain hits – that is, protease, RT, RH and IN domains. In most candidates, hits to a protein domain associated with Gag were found as well. The candidates are consistently linked on the basis of these protein domain clusters, as well as their LTR sequence clusters. ORFs are predominantly longer (up to thousands of bases). We matched these candidates to known *M. domestica* ERV sequences downloaded from Repbase using the *LTRsift* reference matching functionality, resulting in partial and full-length matches to the *ERV2_MD*, *ERV37_MD* and *ERV11_MD* reference sequences. The other, smaller families, containing between 3 and 74 members, in many cases showed full sets of protein domains without being covered by a reference match, suggesting that there may be potential for previously undiscovered or unclassified elements. Some of these protein domains also contain other relevant protein domain hits, such as other Gag domains (Gag_p30, Gag_MA and Gag_p24) or a potential Env/coat polyprotein (TLV_coat).

To assess the possible number of yet undetected full-length candidates, we prepared a filter rule selecting only those candidates with a full set of protein domains. That is, the protease, RT, IN and RH functions must all be represented with at least one pHMM hit associated with that function. We used this rule to select matching candidates from the whole candidate set using *LTRsift*. This delivered a new group containing 1,009 candidates passing the filter. Afterwards we used the reference match coverage filter with threshold 80% to weed out those candidates among the 1,009 that were already matched to a known reference sequence. As a result, we only found 159 candidates with a full set of protein domain hits which have a match to a reference sequence over at least 80% of their internal sequence or more, leaving 850 still unmatched and interesting for further analysis. This illustrates that *LTRsift* allows a user to conveniently prepare interesting subgroups of candidates on the basis of their features.

## Discussion

The advantages of having a specific graphical application for classification, postprocessing and curation of LTR retrotransposon candidates are obvious when compared with a purely manual approach. No data conversion is necessary when using *de novo* candidate detection tools that are able to output the increasingly common GFF3 format to represent their results. *LTRsift* is intended to be used with the *LTRharvest* and *LTRdigest* software that satisfy this requirement.

Once input data are imported into a project file, the project and all associated data are loaded quickly, even when the number of candidates is in the tens of thousands, as may occur when analyzing large mammalian genomes. The window configuration of the user interface, including all open tabs, is saved with the project, allowing the user to continue previously interrupted work in the same environment. *LTRsift* displays candidates and their family memberships in an intuitive way, using common GUI concepts like drag-and-drop to support manipulation. A user familiar with web-based genome browsers can intuitively understand the visual candidate representation. Features are shown in color-coded tracks and are labeled with additional information, such as domain names, match targets, or reading frame and orientation. The view is extensively configurable using a style file, making it possible to adjust both colors and other layout options such as font sizes, as well as enable or disable the display of specific feature types.

Filtering rules are powerful while easy to write with basic programming skills. They support extensive access to the candidate annotation and can be combined to form more complex conditions, not only allowing the user to discard candidates but also to add them to new subgroups that do or do not satisfy the condition described by the filtering rules. Another advantage is that filtering rules are self-contained in one text file per rule. This makes it possible to distribute user-defined filtering rules in the research community.

The matching, clustering and classification components used in *LTRsift* have been designed to be modular, making it possible to quickly add support for new matching tools, clustering strategies or classification algorithms. A unified representation of the candidates as graphs allows the same for other tasks working on annotations and sequences, creating new annotations in the process.

The currently implemented ORF detection and reference annotation components are good examples for such tasks, employing *GenomeTools* functionality and third-party tools like BLAST to transparently extend the annotation within the graphical interface.

We are not aware of any other tool for this exact task of supporting postprocessing and curation of *de novo* LTR retrotransposon annotations in a fashion similar to *LTRsift*. There is a graphical tool, *VisualRepbase*[53], available to display occurrences of TEs, for example, taken from the Repbase Update database [51] in a genomic context together with annotations, for example, downloaded from the NCBI databases. However, *VisualRepbase* does not support the formation of families inside the database, neither does it display the internal structure of the displayed elements in terms of features. By contrast, *LTRsift* does not take the genomic neighborhood of the candidates into account.

The classification approach as described in [5] works well in the *Drosophila* genome. Nevertheless, a large variety of clustering and family assignment strategies exists, and it would be a natural assumption that other, more sophisticated approaches may work better on candidates from other genomes. The modular architecture of *LTRsift* allows the user to incorporate alternative classification strategies in the future, creating a comprehensive and flexible solution for the integration of tools among the diverse landscape of classification methods.

A basic requirement of our classification approach is the presence of annotated internal feature sequences, whose similarities are used to separate candidates into putative families. Many of the candidates satisfy this requirement. Unfortunately, in the *M. domestica* data set no features were detected in the majority of the candidates. Consequently, these candidates can be either false positives or non-autonomous copies. While non-autonomous elements are indisputably important in general, prior analyses have shown that such *de novo* predicted candidates are often unreliable and may well be false positives, as we demonstrated in an earlier use case [5]. However, there are also non-autonomous elements which still retain internal features, which can readily be processed using *LTRsift* and included in a reference sequence set. This set could then be used as a starting point for homology-based detection of more truncated copies in the genome.

World Wide Web-based solutions are becoming increasingly popular for interactive and sometimes distributed analysis of structured data sets due to their platform independence on the user side – only a web browser is needed to access the data from any location with a network connection. We did not follow a web-based approach for the design and development of *LTRsift*. The main reason is that the size of the underlying genome sequences may well become too large to be conveniently uploaded to a web server when analyzing large, for example, mammalian, genomes. By contrast, the size of the annotations is of moderate size (approximately 42 MB for the full unfiltered *M. domestica* candidate set). The sequence is required to perform sequence-based analyses like reference matching, ORF finding or simply to display short motif sequences like PBS and PPT in the candidate details. Hence uploading the annotation alone would not suffice to display every interesting bit of information on the candidates. Instead, we chose to build *LTRsift* on an open source GUI platform intended to run on freely available desktop operating systems. Sharing project files, for example, via a shared network drive, allows a distributed annotation.

While parameterization of the filtering rules is currently only possible by editing the rule files directly, a useful improvement would be the definition of parameter sets, which can then directly be set in the filter selection window. This would also allow the use of multiple instances of the same filter with different parameters without having to copy the file. Another desirable feature would be support for multiple levels of group membership per candidate. For example, a candidate may appear in multiple groups, which in this context describe the candidate on multiple levels: it may appear once in its specific family, and once in a more general ‘copia-like’ group. This could be implemented by assigning tags to candidates and allowing queries on the tags to group candidates together. While functionality to export sequences already exists, it could be very useful to be able to start external tools on given candidate sets (for example, selected candidates or all members of a putative family). A typical example for this kind of use would be a multiple sequence alignment tool such as ClustalX [54] or others. Finally, on some occasions, exporting the linear candidate visualization as an image file would be desirable. Such functionality could easily be implemented as the *AnnotationSketch* engine supports output in a variety of vector and bitmap formats [45].

## Conclusions

We have developed *LTRsift*, a software tool for visualization and postprocessing of *de novo* predicted LTR retrotransposon annotations. It literally allows the user to ‘sift’ through a possibly large quantity of results from a prediction and annotation software like *LTRharvest* and/or *LTRdigest*, which it was designed to work with. However, it relies on standard data formats and can also work on results from other tools, given that the input data are appropriately formatted. LTR retrotransposons can be assigned both automatically and manually to groups considered putative families, which can then serve as a basis for comprehensive sequence library preparation. To the best of our knowledge, *LTRsift* is the first software for this specific task, implementing not only classification but also flexible, customizable filtering in a graphical environment. Relying on a common GUI toolkit from the open source world, the user interface is familiar to everyday computer users. *LTRsift* is efficient enough to allow work with large datasets consisting of up to tens of thousands of candidates on standard desktop hardware, making it likely to be used by life scientists preferring a visual, exploratory hands-on approach to dealing with result data.

## Availability and requirements

• **Project name: ***LTRsift*

• **Project home page:**http://www.zbh.uni-hamburg.de/LTRsift

• **Operating system(s):** UNIX-like systems, for example, Linux or Mac OS X

• **Programming language:** C (GUI software), Lua (filtering rules)

• **Other requirements: ***GenomeTools* version 1.4.2 and higher (not required by the static version), GTK+ 2.2.4 and higher

• **License:** GPL2

## Competing interests

The authors declare that they do not have any competing interests.

## Author’s contributions

StK conceived and guided the project. SaK and SS developed the software. SS performed the example runs. SS and StK wrote the manuscript. All authors read and approved the final version of the manuscript.

## Supplementary Material

Additional file 1 **Technical information.** This PDF file contains additional information on how to write filtering rules for the *LTRsift* software. Besides a description of the rule file structure, it also contains a documentation of the functions to access the representation of the candidate annotation and a documentation of the command line tools. Finally, it lists parameterization details for the example runs. Click here for file

Additional file 2 **Example annotation for *D. melanogaster*.** This gzipped tar archive contains the annotation GFF3 file created as a result of our evaluation runs for the *D. melanogaster* genome, as well as the corresponding sequence in FASTA format. Click here for file
