# Supplementary material for: LTRsift: a graphical user interface for semi-automatic classification and postprocessing of de novo detected LTR retrotransposons
Source: Mob DNA. 2012 Nov 7;3:18. doi: 10.1186/1759-8753-3-18 (PMC3582472; doi:10.1186/1759-8753-3-18)
Supplement: Additional file 1 — Technical information. This PDF file contains additional information on how to write filtering rules for the LTRsift software. Besides a description of the rule file structure, it also contains a documentation of the functions to access the representation of the candidate annotation and a documentation of the command line tools. Finally, it lists parameterization details for the example runs. [file 1759-8753-3-18-S1.pdf]

***LTRsift*: a graphical user interface for semi-automatic classification and postprocessing of *de novo* detected LTR retrotransposons**

Additional File 1

Sascha Steinbiss, Sascha Kastens and Stefan Kurtz

August 30, 2012

Contents

1 Writing filtering rules 1
1.1 Metadata definitions 1
1.2 Input format 2
1.3 Representation of annotations 2
1.4 Storage of cluster and family information 3
1.5 Available data 4

2 Programming interface for feature access 4

3 Parameters used in the example runs 6

4 Command-line tool parameters 9
4.1 The `gt select` tool 9
4.2 The `gt ltrclustering` tool 11

1 Writing filtering rules

1.1 Metadata definitions

Each text file containing a filter rule begins with a few variable assignments specifying some metadata which is displayed in a graphical selection dialog.

```
name        = "Protein Domain Filter"
author      = "Joe User"
version     = "1.0"
email       = "mail@example.org"
short_descr = "Filters out candidates without protein domains"
description = "Filters out a candidate if it does not contain at " ..
              "least one node of type 'protein_match'."
```

Table 1: List of GFF3 fields per feature line. Entries for which the information is not known are specifies by a `.`

Column	Name	Example	Content
1	*seqid*	`seq0`	Sequence region that the feature belongs to.
2	*source*	`LTRharvest`	Free-form qualifier to describe the algorithm or procedure that generated this feature.
3	*type*	`LTR_retrotransposon`	Feature type as Sequence Ontology term.
4,5	*start*, *end*	`1000`, `9000`	1-based start and end position of the annotated feature.
6	*score*	`.`	Score for the feature (such as E-value or p-value)
7	*strand*	`+`	Strand (forward, reverse, both, unknown)
8	*phase*	`0`	Reading frame offset for *CDS* features.
9	*attributes*	`ID=LTR0023`	List of attributes for the current feature, like a unique `ID` or `Name`. `Parent` attributes define a *part-of* relationship with another feature.

Note that "`..`" is the concatenation operator, connecting both description strings in the last two lines in to one string.

1.2 Input format

LTR retrotransposon annotations are input from a file given in the widely used GFF3 format (Generic Feature Format, version 3) [1]. This text-based format defines genomic features and their relationships via a graph-based model in which nodes represent individual features (e.g. LTR, TSD, PBS, ...) and edges represent *part-of* relationships. The latter indicate, for instance, an LTR is *part-of* an LTR retrotransposon, which in turn is *part-of* a repeat region. Available node types and their allowed relationships are – for example – defined in the Sequence Ontology (SO) [2], a standardized set of terms meant to eliminate ambiguity in genomic annotations.

In detail, GFF3 consists of columns separated by tabs, where each line corresponds to a particular feature. That makes it easy to read by humans and also relatively easy to parse by a computer program. The intention for the format was to act as a "lowest common denominator" of annotation formats, thereby allowing easy exchange of annotation information between different software tools.

The first two lines (*meta-lines*) of a GFF file contain the GFF version and the annotated sequence regions. A sequence region could, for example, be a single chromosome or contig. For a sequence region, an identifier (*seqid*) and the range of annotated base positions in this sequence region are given. The following lines describe the features belonging to each sequence region (see table 1). The format also supports additional data like, for example, inline sequences in FASTA format that can be connected to a specific sequence region or cDNA match target.

1.3 Representation of annotations

The filtering rules as described in the main manuscript are a powerful mechanism to identify custom properties in the candidate annotations and to let the *LTRsift* software select matching candidates, e.g. to discard them or to separate them from the rest of the candidates.

The candidate properties are based on the annotation data as given in the input GFF3 file. To fully utilize this functionality, one needs to understand how to access the annotations of the candidates.

In *LTRsift*, one or more annotation files are thus represented by a directed acyclic *feature graph* (Fig. 1).

Each LTR retrotransposon candidate corresponds to a connected component (CC) in the feature graph, referenced by its top-level node (an node without a parent). A CC in this context can be defined as the set of all features which are children of a single top-level node (including the top-level node itself), their children, and so on.

The root node of a feature graph for an LTR retrotransposon must be of type *repeat_region*. The root node has at most three children: two *target_site_duplication* nodes, denoting the 3' and 5' TSDs, and in between an *LTR_retrotransposon* node, the actual element. Note that the TSDs are not required, as there may be repeat identification programs which do not detect them. The *LTR_retrotransposon* node is the main feature of a candidate annotation, with all internal features being children of it. Most prominently, this node must have two children of type *long_terminal_repeat* for the 3' and 5' LTRs. In addition, other internal features are allowed as children of the main node, for example:

- *primer_binding_site* for PBS features, has attributes `tRNA` describing the tRNA and anticodon binding to that PBS, `trnaoffset` to specify the distance from the aligned region on the tRNA from the 3' end, `pbsoffset` to specify the distance from the aligned region on the candidate from the 3' end of the 5' LTR and `edist` to specify the number of mismatches or indels between the PBS and its counterpart on the tRNA,
- *RR_tract* for PPT features,
- *protein_match* for protein domains, has attributes `name` (non-capital "n"!) stating the name of the protein domain model (e.g. Pfam name) and `id` (non-capital letters!) stating an alphanumeric model identifier (e.g. Pfam ID),
- *nucleotide_match* for reference matches or the like, with the usual `target` attribute as defined in the GFF3 specification [1],
- ...

1.4 Storage of cluster and family information

LTRsift makes use of additional information stored in the GFF3 attributes to determine cluster and family membership. The clustering and classification components in *LTRsift* (or their command-line counterparts, see section 4) are designed to set these attributes when processing an annotation. However, when it is desired to use *LTRsift* for the processing of input data not obtained using these tools but other external software, it is required to set these attributes separately. In particular, the following attributes are used:

- For cluster assignment, the respective feature must possess an attribute named `clid` with a numeric cluster ID as the value. Cluster IDs must be unique in the scope of one specific LTR retrotransposon feature (e.g. no two 5' LTRs, 3' LTRs, PBS, protein domain hits for the same profile HMM, etc. may have the same cluster ID). Example:

  ```
  seq0  LTRharvest  RR_tract  10184 10201 . + . Parent=LTR_retrotransposon2;clid=47
  ```
- For family assignment, the top-level *repeat_region* feature must possess an attribute named `ltrfam` with an alphanumeric family ID. This is identical to the family identifier assigned in the *LTRsift* GUI. Family IDs must not contain semicolon characters, as these are used as attribute delimiters in GFF3. Example:

  ```
  seq0  LTRharvest  repeat_region 11271 16798 . ? . ID=repeat_region4;ltrfam=newfam_1
  ```

When loading a GFF file with these attributes set, *LTRsift* will automatically create candidate list columns for the detected features with cluster numbers. Moreover for each family ID encountered in the input file, a new family will be created in *LTRsift* and their members will automatically be placed in it.

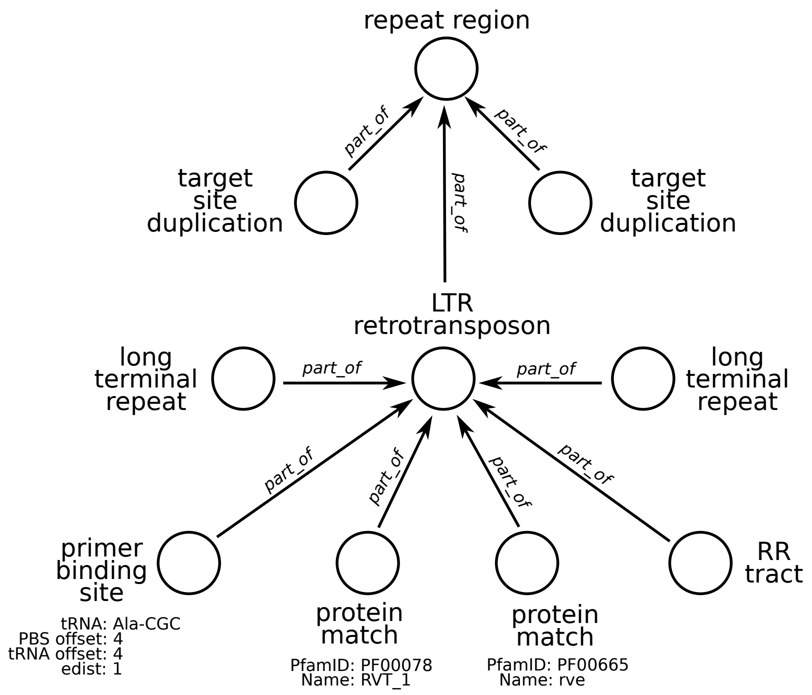

Figure 1: Structure of the LTR retrotransposon feature graph. Nodes represent individual features of a candidate, edges represent *part_of* relationships between them. The direction of nodes is important. The arc is directed towards the feature of which the feature of the other end is a part of. Additional information, such as match details, are stored along with each feature node.

1.5 Available data

The following data are associated with each node in the annotation graph:

- the sequence region (e.g. chromosome, contig, ...) the feature is located on,
- location of the feature on that region in terms of start and end position (1-based),
- strand (forward/reverse),
- a numeric score value (the meaning of which depends on the tool which produced this value, e.g. an E-value),
- a set of key-value pairs containing arbitrary named attributes:
  - feature name/ID (attributes `Name` and `ID`)
  - for PBS: anticodon for PBS-binding tRNAs (attribute `tRNA`, e.g. "Ser-AGA"), number of mismatches and indels in the PBS–tRNA matching (attribute `edist`), offset of the match on the tRNA 3' end (attribute `trnaoffset`), PBS offset from the 5' LTR end (attribute `pbsoffset`)
  - for protein domains: Pfam name or ID for matching pHMMs (attribute `name` and `id`)
  - for LTR retrotransposons: LTR similarity (attribute `ltr_similarity`)

2 Programming interface for feature access

This section describes the interface for accessing the graph-based annotation representation on which the *LTRsift* filtering component is based upon. The programming language used in the filtering rules is basically

Lua (an embeddable scripting language) plus parts of the *GenomeTools* C libraries exported to Lua. For a documentation of Lua itself, its programming language idioms and its standard library programming interfaces, please refer to the Lua reference manual [3]. Also, the complete set of interface definitions (including classes not mentioned here) can be found on the *GenomeTools* web site (http://genometools.org).

`feature_node_new(seqid, type, startpos, endpos, strand)`

Create a new feature node on the sequence with ID `seqid`, type `type` from `startpos` to `end` on strand `strand`. `startpos` and `endpos` always refer to the forward strand, therefore `startpos` has to bo smaller than or equal to `endpos`.

`feature_node:get_strand`

Returns the strand of `feature_node`.

`feature_node:get_source`

Returns the source of `feature_node`.

`feature_node:get_score`

Returns the score of `feature_node`.

`feature_node:get_attribute(attrib)`

Returns the `attrib` attribute of `feature_node`.

`feature_node:get_exons`

Returns an array containing the exons of `feature_node`.

`feature_node:set_source(source)`

Set the source of `feature_node` to `source`.

`feature_node:output_leading`

Show leading part of GFF3 output for `feature_node`

`feature_node:get_type`

Return type of `feature_node` as string.

`feature_node_iterator_new(genome_node)`

Returns a new genome node iterator which performs a depth-first traversal of `genome_node` (including `genome_node` itself).

`feature_node_iterator_new_direct(genome_node)`

Returns a new genome node iterator which iterates over all direct children of `genome_node` (without `genome_node` itself) in a depth-first manner.

`feature_node_iterator:next`

Returns the next genome node in the depth-first traversal for `feature_node_iterator`. If the traversal is complete, the function returns `nil`.

3 Parameters used in the example runs

LTRharvest parameter settings

LTRharvest settings used in the analysis of the *Drosophila melanogaster* and *Monodelphis domestica* sequences. The parameter sets for the *D. melanogaster* analysis are identical to the one used in previous publications [4, 5].

Parameter	*D. melanogaster*	*M. domestica*
LTR length	116–800 bp	100–1000 bp
LTR distance	2280–8773 bp	1000-15000 bp
LTR similarity	91 %	80 %
TSD length	4–20 bp	4–20 bp
motif/TSD search radius	60 bp	60 bp
Seed length	76 bp	30 bp
X-drop X value	7	5
X-drop $score_{mat}$	2	2
X-drop $score_{mis}$	-2	-2
X-drop $score_{ins}$	-3	-3
X-drop $score_{del}$	-3	-3
overlaps	no	best

LTRdigest parameter settings

LTRdigest settings used in the analysis of the *Drosophila melanogaster* and *Monodelphis domestica* candidates.

<table>
<tr><th>Parameter</th><th>D. melanogaster</th><th>M. domestica</th></tr>
<tr><td>PPT search radius</td><td colspan="2">30 bp</td></tr>
<tr><td>PPT length range</td><td colspan="2">8–30 bp</td></tr>
<tr><td>PPT A/G emission probability p_R</td><td colspan="2">0.97</td></tr>
<tr><td>U-box length range</td><td colspan="2">3–30 bp</td></tr>
<tr><td>U-box U/T emission probability p_T</td><td colspan="2">0.91</td></tr>
<tr><td>PBS search radius</td><td colspan="2">30 bp</td></tr>
<tr><td>PBS alignment length</td><td colspan="2">11–30 bp</td></tr>
<tr><td>PBS offset range</td><td colspan="2">0–5 bp</td></tr>
<tr><td>tRNA offset range</td><td>0–40 bp</td><td>0–5 bp</td></tr>
<tr><td>PBS/3’ tRNA end max. edit distance</td><td colspan="2">1</td></tr>
<tr><td>number of tRNAs used</td><td>100 tRNAs</td><td>225 tRNAs</td></tr>
<tr><td>Smith-Waterman $score_{mat}$</td><td colspan="2">5</td></tr>
<tr><td>Smith-Waterman $score_{mis}$</td><td colspan="2">-10</td></tr>
<tr><td>Smith-Waterman $score_{ins}$</td><td colspan="2">-20</td></tr>
<tr><td>Smith-Waterman $score_{del}$</td><td colspan="2">-20</td></tr>
<tr><td>pHMM hit E-value cutoff</td><td colspan="2">Gathering cutoff</td></tr>
<tr><td>max. gap length between chained fragments</td><td colspan="2">50 bp</td></tr>
</table>

tRNA sequences were downloaded from the Genomic tRNA Database [6] and preprocessed to contain each sequence only once.

Profile HMMs used in *D. melanogaster* domain detection

Pfam protein domain models used in the set of LTR retrotransposon/retrovirus-specific domains for the *D. melanogaster* analysis.

Pfam accession#	Pfam ID	Description
PF03732	Retrotrans_gag	Retrotransposon gag protein
PF07253	Gypsy	Gypsy protein
PF00077	RVP	Retroviral aspartyl protease
PF08284	RVP_2	Retroviral aspartyl protease
PF00078	RVT_1	Reverse transcriptase
PF07727	RVT_2	Reverse transcriptase
PF06817	RVT_thumb	Reverse transcriptase thumb domain
PF06815	RVT_connect	Reverse transcriptase connection domain
PF00075	Rnase_H	Ribonuclease H domain
PF00552	Integrase	Integrase DNA binding domain
PF02022	Integrase_Zn	Integrase Zinc binding domain
PF00665	rve	Integrase core domain
PF00098	zf-CCHC	Zinc knuckle domain
PF00385	Chromo	'chromo' (CHRomatin Organisation MOdifier) domain
PF01393	Chromo_shadow	Chromo shadow domain
PF00692	dUTPase	dUTPase domain
PF01021	TYA	TYA transposon protein
PF03078	ATHILA	ATHILA ORF-1 family
PF04094	DUF390	Protein of unknown function (DUF390)
PF08330	DUF1723	Protein of unknown function (DUF1723)
PF04195	Transposase_28	Putative gypsy type transposon
PF05380	Peptidase_A17	Pao retrotransposon peptidase

Profile HMMs used in *M. domestica* domain detection

Pfam protein domain models used in the *Monodelphis domestica* analysis.

Pfam accession#	Pfam ID	Description
PF01140	Gag_MA	Matrix protein (MA), p15
PF02337	Gag_p10	Retroviral GAG p10 protein
PF01141	Gag_p12	Gag polyprotein, inner coat protein p12
PF00607	Gag_p24	gag gene protein p24 (core nucleocapsid)
PF02093	Gag_p30	Gag P30 core shell protein
PF00692	dUTPase	dUTPase domain
PF00077	RVP	Retroviral aspartyl protease
PF00078	RVT_1	Reverse transcriptase
PF07727	RVT_2	Reverse transcriptase
PF13456	RVT_3	Reverse transcriptase-like
PF13456	RVT_N	N-terminal domain of reverse transcriptase
PF06817	RVT_thumb	Reverse transcriptase thumb domain
PF06815	RVT_connect	Reverse transcriptase connection domain
PF00552	Integrase	Integrase DNA binding domain
PF02022	Integrase_Zn	Integrase Zinc binding domain
PF00665	rve	Integrase core domain
PF00075	Rnase_H	Ribonuclease H domain
PF00429	TLV_coat	ENV polyprotein (coat polyprotein)
PF08791	Viral_env	Viral envelope protein
PF09590	Env-gp36	Env-gp36 lentivirus glycoprotein
PF03408	Foamy_virus_ENV	Foamy virus envelope protein
PF00516	GP120	Envelope glycoprotein GP120
PF03056	GP36	Env gp36 protein (HERV/MMTV type)
PF00517	GP41	Envelope Polyprotein GP41
PF00098	zf-CCHC	Zinc knuckle domain
PF13966	zf-RVT	zinc-binding in reverse transcriptase

4 Command-line tool parameters

Note that use of these commands require that the *GenomeTools* software package (version 1.4.2 or later) [7] is installed on the target system, and that the `gt` executable can be found in an accessible path.

4.1 The `gt select` tool

The `gt select` tool subsumes the *LTRsift* filtering functionality as a command line tool. It has the following syntax:

```
gt select [option ...] [GFF3_file ...]
```

The GFF3 data to filter can be given either as a list of files appended to the options (see Table 2) or – if no file names are given – GFF3 data is read from standard input.

Table 2: Options of the `gt select` tool.

`-seqid`	select feature with the given sequence ID (all comments are selected), default: undefined
`-source`	select feature with the given source (the source is column 2 in regular GFF3 lines), default: undefined
`-contain`	select all features which are contained in the given range, default: undefined
`-overlap`	select all features which do overlap with the given range, default: undefined
`-strand`	select all top-level features(i.e., features without parents) whose strand equals the given one (must be one of `+-.?`), default: undefined
`-targetstrand`	select all top-level features (i.e., features without parents) which have exactly one target attribute whose strand equals the given one (must be one of `+-.?`), default: undefined
`-targetbest`	if multiple top-level features (i.e., features without parents) with exactly one target attribute have the same target_id, keep only the feature with the best score. If -targetstrand is used at the same time, this option is applied after -targetstrand. Memory consumption is proportional to the input file size(s), default: no
`-hascds`	select all top-level features which do have a CDS child, default: no
`-maxgenelength`	select genes up to the given maximum length, default: undefined
`-maxgenenum`	select the first genes up to the given maximum number, default: undefined
`-mingenescore`	select genes with the given minimum score, default: undefined
`-maxgenescore`	select genes with the given maximum score, default: undefined
`-minaveragessp`	set the minimum average splice site probability, default: undefined
`-rule_files`	specify Lua files to be used for selection
`-rule_logic`	select how multiple Lua files should be combined choose from AND—OR, default: AND
`-dropped_file`	save non-selected features to file, default: undefined
`-v`	be verbose, default: no
`-o`	redirect output to specified file, default: undefined
`-gzip`	write gzip compressed output file, default: no
`-bzip2`	write bzip2 compressed output file, default: no
`-force`	force writing to output file, default: no
`-help`	display help and exit
`-version`	display version information and exit

A wide variety of filtering criteria exists – besides the Lua-based rule functionality (-rule_files and -rule_logic) it is also possible to select features by length, location, strand, and so on.

Note that this tool *selects* files instead of *filtering* them away, acting like a negated version of what *LTRsift* does.

4.2 The `gt ltrclustering` tool

The `gt select` tool implements the clustering and classification functionality from *LTRsift* as a command line tool. It has the following syntax:

```
gt ltrclustering [option ...] indexname [GFF3_file ...]
```

The options are given in Table 3. Again, the GFF3 data with the candidates can be given either as a list of files or as standard input. The `indexname` is the name of the encoded sequence file from which the candidates were predicted, minus the file suffixes (`.esq`, `.des`, etc.).

Table 3: Options of the `gt ltrclustering` tool.

-psmall	specify how many percent of the smaller sequence a match needs to cover in order to cluster the two sequences of the match. default: 0
-plarge	specify how many percent of the larger sequence a match needs to cover in order to cluster the two sequences of the match. default: 0
-o	redirect output to specified file default: undefined
-gzip	write gzip compressed output file default: no
-bzip2	write bzip2 compressed output file default: no
-force	force writing to output file default: no
-help	display help and exit
-version	display version information and exit

References

[1] Stein L: **Generic Feature Format Version 3** [http://www.sequenceontology.org/gff3.shtml].

[2] Eilbeck K, Lewis S, Mungall C, Yandell M, Stein L, Durbin R, Ashburner M: **The Sequence Ontology: A Tool for the Unification of Genome Annotations**. *Genome Biology* 2005, **6**(5):R44, [http://genomebiology.com/2005/6/5/R44].

[3] Ierusalimschy R: *Programming in Lua, Second Edition*. Lua.Org 2006.

[4] Ellinghaus D, Kurtz S, Willhoeft U: **LTRharvest, an efficient and flexible software for de novo detection of LTR retrotransposons**. *BMC Bioinformatics* 2008, **9**:18, [http://www.biomedcentral.com/1471-2105/9/18].

[5] Steinbiss S, Willhoeft U, Gremme G, Kurtz S: **Fine-grained annotation and classification of *de novo* predicted LTR retrotransposons**. *Nucleic Acids Res.* 2009, **37**:7002–7013, [http://nar.oxfordjournals.org/cgi/content/full/37/21/7002].

[6] Chan PP, Lowe TM: **GtRNAdb: a database of transfer RNA genes detected in genomic sequence**. *Nucleic Acids Res.* 2009, **37**:D93–97.

[7] Gremme G, Steinbiss S, Kurtz S: **GenomeTools** . *http://genometools.org* 2012.
